# How Weird is The Worm? Evolution of the Developmental Gene Toolkit in *Caenorhabditis elegans*

**DOI:** 10.3390/jdb7040019

**Published:** 2019-09-28

**Authors:** Emily A. Baker, Alison Woollard

**Affiliations:** Department of Biochemistry, University of Oxford, South Parks Rd, Oxford OX1 3QU, UK; emily.baker@spc.ox.ac.uk

**Keywords:** gene toolkit, evo-devo, gene duplication

## Abstract

Comparative developmental biology and comparative genomics are the cornerstones of evolutionary developmental biology. Decades of fruitful research using nematodes have produced detailed accounts of the developmental and genomic variation in the nematode phylum. Evolutionary developmental biologists are now utilising these data as a tool with which to interrogate the evolutionary basis for the similarities and differences observed in Nematoda. Nematodes have often seemed atypical compared to the rest of the animal kingdom—from their totally lineage-dependent mode of embryogenesis to their abandonment of key toolkit genes usually deployed by bilaterians for proper development—worms are notorious rule breakers of the bilaterian handbook. However, exploring the nature of these deviations is providing answers to some of the biggest questions about the evolution of animal development. For example, why is the evolvability of each embryonic stage not the same? Why can evolution sometimes tolerate the loss of genes involved in key developmental events? Lastly, why does natural selection act to radically diverge toolkit genes in number and sequence in certain taxa? In answering these questions, insight is not only being provided about the evolution of nematodes, but of all metazoans.

## 1. Introduction

Evolutionary developmental biology aims to explain how the modification of developmental processes and mechanisms over the course of evolution produces changes in animal morphology. While the model nematode *Caenorhabditis elegans* has made vital contributions to our understanding of the molecular mechanisms of development, its role in illuminating the evolution of developmental processes has for the most part been limited to comparative analysis between different *Caenorhabditis* species and a few more distantly related nematodes, such as *Pristionchus pacificus*, and more recently between geographically distinct populations, or wild isolates, of the worm itself [1].

Traditionally, the field of *C. elegans* developmental genetics has focussed on particular aspects of development, for example sex determination [2], neurobiology [3], and vulval organogenesis [4]. Such studies have been expanded in recent years to other *Caenorhabditis* species, marking the dawn of nematode comparative developmental biology. However, insights garnered from studies using these so-called ‘satellite’ systems [5] have arguably been limited by the gravitational pull of the *C. elegans* paradigm [6], restricting their scope and interpretation. Thus, the current approach has been largely descriptive. This is a missed opportunity, because evolutionary developmental biology is more than merely describing how development is controlled in model and non-model systems. Moving beyond asking ‘how’ development occurs in different species, studies are now being performed with the central focus of asking ‘why’ they do so, and to what evolutionary outcome.

By contrast, evolutionary developmental biology research performed in other non-nematode systems has concentrated on uncovering the gene regulatory networks responsible for orchestrating the developmental programmes underlying animal diversity [6]. The conserved set of genes present in all bilaterians—known as the gene toolkit—has ancient roles in body axis specification and cell fate determination during development [7], as well as being repeatedly co-opted for a variety of taxon-restricted novel phenotypes [8,9,10]. Toolkit genes include many familiar signal transduction pathways such as Hedgehog, Wnt, and Notch, and transcription factors such as homeobox genes [6], which together orchestrate development in all bilaterian lineages. An important driver in toolkit evolution is gene duplication. Aside from providing raw material upon which selection can act, an idiosyncratic pattern of duplication and loss of toolkit genes in a given taxon has the potential to give each species a unique gene toolkit [11,12].

Far from being an archetypal bilaterian with respect to its gene toolkit, *C. elegans* has evolved a divergent suite of toolkit genes, both in terms of their copy number variation and lack of sequence conservation compared to their arthropod and mammalian orthologues. In the second part of this review, we explore what the effect of evolving a divergent gene toolkit has been on *C. elegans* development. We also consider what this has meant for the worm as a model in evolutionary developmental biology, and what being an atypical bilaterian has to offer the field of evolutionary developmental biology at large.

## 2. The Nematode Phylum: Beyond ‘The Worm’

*Caenorhabditis elegans* is a well-established model organism for the study of development, genetics, and genomics, as well as seemingly ever-expanding fields of related research. Numerous qualities enabled it to thrive as a genetic and developmental model system from the mid-1970s onwards [13], including its small size, relatively simple anatomy, and nervous system, as well as its rapid lifecycle and transparency from egg to adult. In addition, *C. elegans* populations consist mostly of self-fertilising hermaphrodites with the occasional spontaneous male, facilitating genetic analysis.

As *C. elegans* became increasingly utilised by biologists, a plethora of resources became available that made ‘The Worm’, as it is affectionately known, an even more convenient system with which to answer biological questions. Most notably, the entire invariant cell lineage was traced throughout development [14], and later the whole genome was sequenced: the first complete genome of a multicellular animal [15]. Consisting of 100 Mb and encoding 20,190 protein-coding genes (WormBase Release WS270), the worm’s compact genome represents an invaluable tool for tackling the complex, multifaceted nature of biological problems.

**Figure 1:**
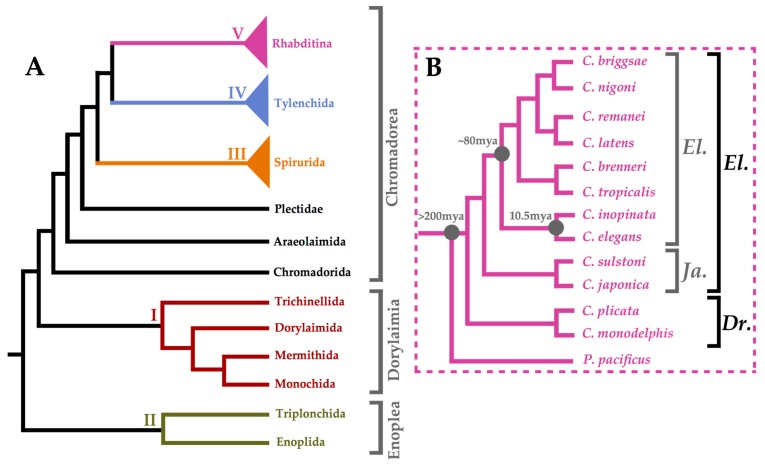
**Phylogenetic Summary of Nematoda. Panel** (**A**) shows a dendrogram summarising the results of Blaxter et al.'s maximum parsimony analysis as well as the maximum likelihood analysis performed by Smythe et al. on nematode orders [18,19]. Clades I–V are colour-coded: I (burgundy), II (olive), III (orange), IV (light blue), and V (purple). Panel (**B**) shows a summary dendrogram of the *Caenorhabditis* genus adapted from Bayesian analysis by Stevens et al. [22]. All species are in the *Caenorhabditis* genus and rooted with a *Pristionchus pacificus* outgroup. The *Elegans* (*El.*) and *Drosophilae* (*Dr.*) supergroups are depicted (black) as well as the *Elegans* (*El.*) and *Japonica* (*Ja.*) groups (grey) therein.

Following the post-genomic era, high-quality genome assemblies became available for many species from across the nematode phylum (Figure 1A) [16,17,18,19,20]. Nematoda is further divided into three main lineages, namely, Enoplea, Dorylaimia, and Chromadorea, although orders are commonly organised into five major clades that do not correspond to the divisions of classical taxonomy [18]. Clade V nematodes—including *C. elegans* and other Rhabditina—have been sequenced the most extensively. Non-parasitic, soil-dwelling bacterivorous members of this suborder offer many of the same advantages as *C. elegans* in terms of being tractable experimental systems [19,20]. Figure 1B summarises the phylogenetic relationship between particular Clade V species. *C. inopinata* is the recently discovered sister species of *C. elegans* [21]. Its genome is larger than that of *C. elegans* at 123 Mb, which is thought to be due to highly expanded transposable elements, yet it has lost many protein-coding genes such as those in chemoreceptor gene families [21]. The phylogenetic position of these species, together with their evolutionarily conserved morphology, and the ease with which they can be cultured under laboratory conditions, make *Pristionchus pacificus*, *C. japonica*, *C. inopinata*, *C. brenneri*, *C. remanei,* and *C. briggsae* prime comparative systems for the *C. elegans* model.

Central themes of *C. elegans* research have been investigated in these satellite systems [23]. From events occurring during early embryogenesis [24] and primary sex determination [23,25], to post-embryonic events such as vulval development [23,26,27] and neurogenesis [28,29], deviations from the *C. elegans* paradigm have been meticulously catalogued in many nematode species. This characterisation of developmental variation in a phylogenetic context among nematodes has previously been reviewed by Eric Haag and colleagues [23], and so will not be described in this work. However, this work, as we shall see, provides an essential platform for interrogating the more open questions in nematode evolutionary developmental biology.

## 3. Phyletic Body Plans and the Phylotypic Stage

It is striking that one of the most species-rich bilaterian phyla displays the least morphological diversity. For the majority of nematode species, the lack of variation in their body plans is explained by the similarity of their developmental programmes at both the cellular and molecular level [24,30,31,32]. *C. elegans*, *C. brenneri*, *C. remanei*, and *C. briggsae* have nearly identical patterns of cell migration, cell death, and differentiation during embryogenesis [30]. In all four *Caenorhabditis* species, the same cells contribute to the same tissues; thus, the conservation in lineage translates into a conservation of cell fate. This implies that the programmes specifying the nematode body plan have remained unchanged for approximately 80 million years of evolution. It is extraordinary to consider that these four nematode species display more genomic divergence from each other than humans and mice do, yet are almost identical morphologically [16,31]. 

Michal Levin and colleagues uncovered the molecular basis for this apparent paradox using comparative transcriptomics [32]. The transcriptional profiles of *C. japonica*, *C. elegans*, *C. brenneri*, *C. remanei*, and *C. briggsae* embryos throughout development reveal that all species show a rapid burst of transcriptional activity at ventral closure [32]. Notably, ventral closure is not chronologically aligned in all species, indicating that it is a property of this stage that is coupled to its transcriptional activity, rather than the pattern simply being due to the amount of time elapsed.

The transcriptomes of *Caenorhabditis* embryos undergoing ventral closure are enriched for developmental regulators involved in tissue differentiation, including transcription factors such as homeobox genes (*mab-5*, *ceh-24,* and *ceh-30*) and T-box genes (*tbx-43*). Subsequent analyses showed that many of the genes upregulated during ventral closure give rise to loss-of-function phenotypes in *C. elegans* upon knockdown by RNAi [32,33]. Importantly, stages other than ventral closure were not associated with enrichment for genes that produced similar, severe, developmental phenotypes. Furthermore, orthologues of the abundant transcripts present during nematode ventral closure are similarly upregulated throughout two temporally distinct stages of arthropod embryogenesis [34], and in the tailbud and neurulation stages of the amphibian *Xenopus tropicalis* [35]. It is known that these stages in flies and frogs correspond to the phylotypic period in chordates [36]; that is the time during the middle of metazoan development when animals most closely resemble all other species [37], and so highly conserved pattern determinants dominate their transcriptomes [38]. As such, it is likely that two disparate developmental milestones became coupled in nematodes, which may at least partially explain the limited morphological diversity observed in Nematoda compared to other large bilaterian phyla, such as Arthropoda and Chordata. More broadly, this work also suggests that coupling and elaborating on embryonic processes might be a strategy for evolving animal body plans. 

The aforementioned analyses used members of the *Caenorhabditis* genus to show that interspecific morphological similarity is underpinned by conserved molecular profiles that give rise to the same patterns of cellular behaviour throughout development; but what about nematode species that develop differently? Enopleans, for example *Tobrilus stefanskii*, exhibit patterns of embryonic cleavage more similar to the tardigrade *Hypsibius dujardini* than cleavage in *C. elegans* [24]. Even among chromadoreans, variation exists in many aspects of embryogenesis [24,39]. There are two types of early cleavage in enopleans and four in chromadoreans, all of which are due to alterations in the activity of subcellular structures known as Polarity Organizing Centers (POCs) that orientate cleavage spindles in founder cells along the anteroposterior axis in response to the changing distribution of partitioning (PAR) proteins [24,40]. The type of embryonic cleavage in *C. elegans* is representative of most chromadoreans, and all other cleavage patterns can be explained by modifications in the interplay of PAR proteins and POCs. However, this is perhaps where *C. elegans* ceases to be representative of its phylum, as derived nematode clades have evolved increased numbers of somatic founder cells, and in doing so have come to rely upon an invariable, or lineage-dependent, means of cell fate specification, as opposed to a variable, or position-dependent means. Thus, while all nematode species must pass through the same developmental milestones to generate the same body plan, cellular behaviour leading to these is not always conserved, and so it can be said that there are many ways to make a worm.

## 4. Tools to Make a Worm

**Figure 2:**
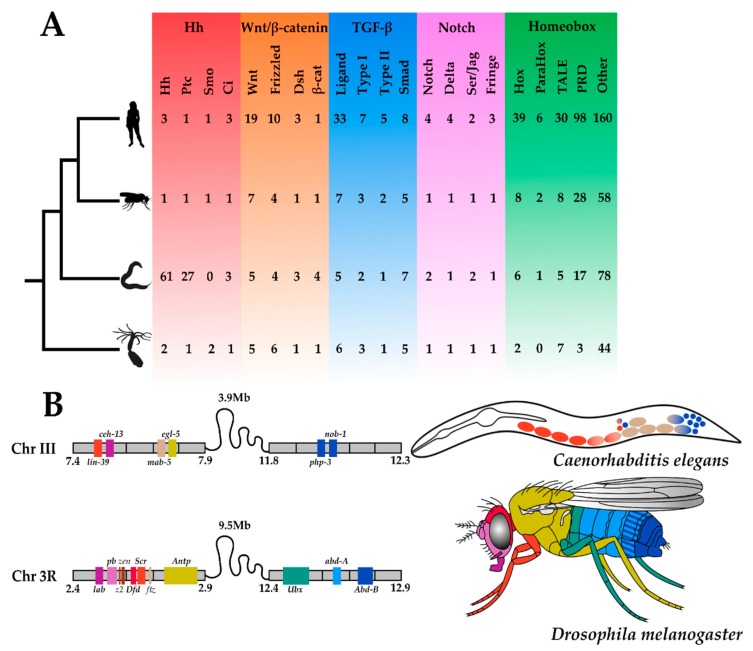
**Metazoan Toolkit Evolution. Panel** (**A**) depicts the phylogenetic relationships between four metazoan species, from the top: *Homo sapiens*, *Drosophila melanogaster*, *Caenorhabditis elegans*, and *Nematostella vectensis*. Silhouettes of animals were obtained from http://phylopic.org (open source). Their gene toolkits, from the left. Hedgehog (red): Hedgehog (Hh), Patched (Ptc), Smoothened (Smo), Cubitus Interruptus (Ci); Wnt (orange)-*sensu stricto*: ‘Wingless-related Integration site’ (Wnt), Frizzled, Dishevelled (Dsh), β-cat (β-catenin); Transforming Growth Factor-β (TGF-β) (blue)-*sensu lato*: Ligand, Type I receptor, Type II receptor, Smad (Small Mothers Against Decapentaplegic); Notch (purple)-*sensu stricto*: Notch, Delta, Serrate/Jagged (Ser/Jag), Fringe; Homeobox (green): Hox, ParaHox, TALE (Three Amino Acid Loop Extension), PRD (Paired), Other (all other additional homeobox genes mined from the proteomes); are depicted. All copy numbers were obtained by the authors of this review from mining the predicted proteomes of *H. sapiens*, *D. melanogaster*, *C. elegans* and *N. vectensis* using reciprocal (NCBI) BLASTp searches. Panel (**B**) depicts the Hox clusters of *C. elegans* and *D. melanogaster*. Their genomic location with an approximate scale (Mb = Megabase) and syntenic relationships are schematised, where gene colours denote Hox orthogroups. These colours are also used to depict the gene expression in the adults of these two species.

Investigating *C. elegans* development in concert with other nematodes is important for understanding the evolution of the phylum, but in order to unearth the molecular principles underpinning the evolution of development, *C. elegans* and other nematodes must be contextualised within the animal kingdom. In the post-genomic era, animal phyla are compared based on their gene toolkits: the small subset of genes in an organism’s genome that controls its development (Figure 2). Analysing the functions of many components in the *C. elegans* gene toolkit has been critical for understanding their involvement in the development of other animals, for example the conserved role of TGF-β signalling in specifying cellular identity. 

Gene duplications and gene losses have shaped the gene toolkits of animals over the course of evolution [41,42]. The balance between these two dynamic processes not only accounts for copy number differences between species, but also aspects of their morphological diversity as found in studies of moths, molluscs, and mammals [42]. The gene toolkit has become the ‘Top Trumps’ of the animal kingdom, where numeric comparisons enable the easy identification of animal oddities from more typical bilaterians. While it can be argued that all species are animal oddities in a certain aspect of their gene toolkit, *C. elegans* can be considered atypical with respect to two major components, namely, the Hedgehog pathway and the Hox cluster, both of which exhibit unusual taxon-specific patterns of gene family evolution. 

### 4.1. Gaining Tools: The Hedgehog Pathway

Amongst the small suite of signalling pathways in the gene toolkits of animals is the Hedgehog pathway (Figure 2A), which is essential for many aspects of metazoan development [43,44]. From segment polarity determination in arthropods and annelids [45] to the development of the vertebrate notochord [42], the role of Hedgehog signalling in the bilaterians has been well-characterised. Although the role of Hedgehog in cnidarians is currently unknown, a complete pathway exists in *Nematostella vectensis* and other species, which implies it has an early metazoan origin [46,47]. The Hedgehog gene has diversified little throughout the Bilateria, with most species possessing only one true orthologue. The genome expansion in vertebrates has given rise to three ohnologues (paralogues derived from whole genome duplication) in amniotes, and due to an additional round of whole genome duplication, four or five ohnologues in ray-finned fish [48]. 

Thomas Bürglin and colleagues attempted to characterise the Hedgehog ligands in *C. elegans* by mining the genome for a Hedge domain, that is the N-terminal portion of Hedgehog responsible for signalling [49,50]. After noting a surprising absence of hits upon searching for the Hedge domain, multiple ORFs were found only on the basis of sequence similarity to the Hog domain—that is the C-terminus, which is typically degraded prior to secretion of the processed Hedgehog. This led to the classification of novel N-terminal domains associated with Hog in *C. elegans*, initially Warthog (WRT) and Groundhog (GRD) [50], followed by Ground-like (GRL) and Quahog (QUA) [51].

Despite the radiation of these Hedgehog-related (Hh-r) gene families and the expansion of the putative receptors known as Patched and the Patched-related (Ptr) genes, there is no canonical Hedgehog pathway in nematodes [50,51,52,53,54]. *C. elegans* and other chromadoreans do not possess bona fide Hedgehog orthologues, and both major classes of nematodes lack Smoothened homologues, the G-protein coupled receptor that is inhibited by Patched in the absence of Hedgehog. In this way, the evolution of the Hedgehog pathway in nematodes is a story of both gene gain and loss. The loss of the Hedgehog pathway has likely prevented the evolution of many morphological features that are associated with the pathway in other bilaterian phyla, such as segmentation and a complex nervous system, both of which require the positional information provided by Hedgehog signalling. Additionally, it could be seen as no coincidence that transitioning towards a totally lineage-dependent means of cell fate specification during embryogenesis occurred simultaneously with the loss of Hedgehog during nematode evolution. It should be noted that *Trichinella spiralis* and some other enopleans have retained a true Hedgehog homologue, but it is unknown if this is required for their more position-dependent mode of embryogenesis. In any case, the loss of Hedgehog proper is met with the evolution of an alternative repertoire of Hh-r genes that have facilitated the evolutionary innovation in *C. elegans*. 

Taxon-restricted genes are now widely recognised as sources of genetic material that natural selection often uses to generate novel functions in an array of animal taxa [55,56,57]. Many taxon-restricted genes are lineage-specific paralogues, but they may also arise de novo from non-coding sequences [58,59]. One such taxon-restricted gene in *P. pacificus* is *self-1*, which is an ‘orphan’ gene that is essential for the kin recognition system, enabling this predatory nematode to avoid cannibalism, while promoting the killing of competing nematodes [60]. In total, taxon-restricted genes account for 10%–30% of eukaryotic genomes, and they are increasingly implicated in the phenotypic innovations that have occurred throughout nematode evolution [59]. 

The Warthogs are 10 taxon-restricted genes in the Hh-r superfamily [50]. Only one family member has pseudogenised, while the remaining nine have specialised in various aspects of post-embryonic development, including: cell fate determination in vulval organogenesis, body size regulation, and the generation of left-right asymmetry in the middle bodies of late larval stage worms [61]. It is less certain what roles the other Hh-r superfamily members play in *C. elegans*, but it is known that many of these gene families, including the Warthogs, are involved in ecdysis [51,53,61,62,63]. Therefore, according to the classical model of paralogous gene evolution [64], Hh-r genes are derived from an ancestor that was principally involved in ecdysis, and following their inception, have subfunctionalised, while concomitantly neofunctionalising in other aspects of post-embryonic development [61]. 

The Patched and Ptr genes display no overlaps with the neofunctions of the Warthogs in *C. elegans*. There are two Patched genes, one Patched pseudogene, and 24 Ptr genes [65]. *ptc-1* is expressed in oocytes and involved in cytokinesis [65] and *ptr-7* is necessary for endocytosis during tubulogenesis [66]. Similar to the Hh-r genes, the Patched and Ptr families are also involved in ecdysis, implying they were at least ancestrally in the same signalling pathway [53,62,63].

While none of the Hh-r or Ptr genes are thought to be involved in sex determination, the Gli/Ci orthologue, *tra-1,* is well-known as the terminal global regulator in a cascade of sex-determining genes in nematodes [2,67]. In governing all aspects of somatic sexual differentiation and germline differentiation, the *tra-1* locus shapes the sex ratio of worm populations, which affects every aspect of their ecology and evolution [67]. Thus, in losing critical components and thereby dissolving the Hedgehog pathway, natural selection has utilised the remaining genetic material to create phenotypic novelty in *C. elegans*, either by expanding ancestral toolkit genes or repurposing conserved elements. 

### 4.2. Losing Tools: The Hox Complement

Hox genes are a set of homeobox transcription factors that are responsible for patterning the anteroposterior axis. The *Drosophila* Hox complement is split into two clusters on the same chromosome, yet crucially, all genes are spatially colinear with respect to gene expression (Figure 2B). Rebuilding the ancestral protostome Hox cluster from the gene complements in extant phyla suggests there were at least nine orthologous groups that have retained their genomic organisation in many species [68,69]. Long thought to be highly conserved, tightly clustered and colinear in all animals, the genomic organisation of the Hox complement can occasionally come in alternative flavours [70].

The Hox cluster is neither conserved nor clustered in *C. elegans* [68,69,70,71]. Instead, *C. elegans* has a depauperate and dispersed gene complement that is the result of a gradual loss of Hox genes throughout its evolutionary history. With only six Hox genes—the anterior gene *ceh-13*; two central genes, *lin-39* and *mab-5*; and three posterior genes, *egl-5*, *php-3*, and *nob-1*—the *C. elegans* Hox complement is organised in three pairs distributed across 5 Mb of chromosome III [63,64]. Most nematode species have slight variations on the *C. elegans* Hox cluster. Enopleans and early branching chromadoreans often have an additional *antennapedia*-like gene, *ant-1*, while more derived taxa have a similar or even a more depauperate Hox repertoire compared to *C. elegans* [68,69,71]. The atomised Hox complement in nematodes is also found in some cnidarians [72], tunicates [73], and flatworms [74], but few phyla display such consistent deviation from the classical organised Hox cluster as in Nematoda.

Nematomorpha is the sister phylum to Nematoda, and its species share a simple vermiform body plan. Unlike nematodes, nematomorphs have retained the ancestral protostome Hox genotype [68], and so the degeneration of the Hox cluster in nematodes cannot simply be a consequence of evolving a vermiform body plan. Thus, it is worth considering whether the evolution of development in nematode worms has been affected by the degeneration of this gene class that is considered fundamental to generating the bilaterian body plan. 

Similar to in vertebrates and arthropods, Hox genes in *C. elegans* have roles in specifying cell fates along the body axis, and their expression broadly follows spatial collinearity by orthologue group. However unlike in other bilaterians, Hox gene expression in *C. elegans* is lineage-dependent, and only the anterior gene *ceh-13* [75] and the posterior genes *php-3* and *nob-1* are required for embryogenesis [76]. Surprisingly, worms carrying a null mutation in the more divergent *nob-1* have extreme posterior-to-anterior cell fate transformations, yet the loss-of-function phenotypes of *php-3* (which is highly conserved in other bilaterians) are merely minor posterior patterning defects. The other three Hox genes are not essential for embryogenesis, but are required for cell fate determination and cell migration in aspects of post-embryonic development, such as in the formation of the male tail (*mab-5*) and vulval organogenesis and function (*lin-39* and *egl-5*, respectively) [77].

It is rare for protostome Hox genes to dispense with their ancestral roles and evolve entirely taxon-specific functions. Therefore, while the deterioration of the nematode Hox cluster may not have directly given rise to their simple body plan, which lacks segments and a complex nervous system, the relaxed selection on nematode Hox genes may have permitted their exploitation of these new and unusual, post-embryonic functional niches.

Prior to the Cnidaria/Bilateria split during the evolution of the urbilaterian ancestor, the Hox cluster duplicated, and the divergence of genes contained therein ensued [78]. Nematodes have since lost all but one of the three members of the ParaHox cluster, known as *caudal (cad/Cdx)*, or *pal-1*. *caudal* orthologues are essential for the formation of posterior structures by regulating Hox gene expression in flies, vertebrates, and *C. elegans* [79,80]. *pal-1* is required for the expression of *mab-5*, and can be suppressed by *sop-2* [81]. *sop-2* loss-of-function in a wild-type background causes the ectopic expression of multiple homeobox genes, including *mab-5* and *pal-1*, in diverse tissue types. SOP-2 has a sterile α-motif (SAM) domain that is also present in Polycomb group (PcG) chromatin-regulating proteins, which are required for the negative regulation of Hox gene expression in vertebrates and flies [81,82]. Taken together, these data imply that irrespective of Hox and ParaHox gene loss in *C. elegans*, the remaining orthologues have homologous mechanisms of repression, despite their extensive functional divergence. So, the biochemical signature of Hox and ParaHox regulation is said to be ancient and conserved even in diverse, extant bilaterian taxa.

Little is known about the remaining homeobox genes in *C. elegans* [83]. Despite having fewer homeobox genes than flies, *C. elegans* has approximately 25 homeobox genes that do not fall into any of the existing homeobox gene classifications, and are known to be rapidly evolving [84]. Unusually, many of these genes encode multiple homeodomains [83,85]. A family of fast evolving, eutherian-specific homeobox genes have been implicated in blastocyst formation in mice [86,87], which is an early developmental event occurring exclusively in mammals. Therefore, it is possible that this burgeoning of nematode-specific homeobox genes—many of which are found only in more derived chromadoreans—are in some way contributing to the unique developmental programme that is so distinctive to Nematoda.

### 4.3. Changing Tools: Wnt and Notch Signalling

Large-scale gene duplications and gene losses have played major roles in the evolution of nematode development, but more subtle changes to the gene toolkit of *C. elegans* have had important consequences on worm development, too. A couple of gene duplications in key components of the Wnt and Notch signal transduction pathways have preceded noteworthy patterns of sequence evolution in these genes, which have striking parallels with mammalian toolkit evolution. 

Wnt signalling (Figure 2A) controls the development of animals across the metazoan tree of life [88]. As important regulators of *C. elegans* development, Wnts function in processes as diverse as cell fate specification, asymmetrical division, cell migration, and synapse formation [89]. The canonical Wnt pathway controls the transcription of target genes through a terminal effector protein, β-catenin. While having relatively typical copy numbers of all other Wnt pathway components, the *C. elegans* genome encodes four β-catenin-like proteins (Figure 2A). These are SYmmetrical Sister cell hermaphrodite gonad defect (SYS-1), Worm aRMadillo (WRM-1), Beta-catenin/Armadillo Related (BAR-1), and HuMPback (dorsal lumps) (HMP-2), which share a percentage sequence similarity with human β-catenin of 9%, 19%, 25%, and 29%, respectively [90].

Ostensibly divergent and far from a canonical β-catenin in primary sequence, at least one of these paralogues is now known to be structurally almost identical to human β-catenin [90]. The crystal structure of the SYS-1 protein shows that it possesses 12 armadillo repeats that stack upon one another to form a superhelix, similar to human and fly β-catenin. The complex that SYS-1 forms with the T cell factor (TCF) transcriptional coactivator, POsterior Pharynx defect (POP-1), is nearly identical to the canonical β-catenin/TCF complex; perhaps most importantly, the two proteins in both complexes are anchored by a conserved aspartate to the lysine salt bridge [90,91]. Confirming the biological relevance of this in vivo by inducing a point mutation in *pop-1* changes the aspartate to a glutamate in the salt bridge, which abrogates POP-1 function. It is unknown if the three-dimensional conformation of SYS-1 and human β-catenin is conserved in the nematode phylum or beyond, as homologues are routinely recognised by their primary sequence without structural determination. Traditional sequence comparisons between taxa in evolutionary developmental biology miss such instances of structural homology; thus, the remarkable structural similarity between human and nematode β-catenin in spite of extensive sequence divergence is a unique story that is rarely described, but has probably occurred many times over the course of evolution. This highlights the potential for different approaches to uncover the molecular evolution of developmental pathways, and illustrates the true insight that can be gained from comparing gene toolkits.

The three-dimensional structure of SYS-1 retrospectively illuminates its previously characterised role as a β-catenin-like coactivator, yet SYS-1 has never been found to play a β-catenin-like role in adherens junctions for cell–cell adhesion, in which HMP-2 is known to be involved [92,93]. This implies that the *C. elegans* β-catenin paralogues have taken on aspects of the ancestral gene’s function, whereas these roles are still carried out by a single gene in other extant taxa. However, the evolution of the nematode β-catenin family members is more nuanced than mere subfunctionalisation [94]. In fact, only BAR-1 participates in the canonical signalling pathway, as the majority of Wnt signalling that occurs during *C. elegans* development is considered non-canonical, and part of a highly derived Wnt/β-catenin asymmetry pathway in which SYS-1 and WRM-1 have neofunctionalised to collaborate with additional components in innovative ways [94]. In the Wnt/β-catenin asymmetry pathway, target gene activation requires WRM-1 and the mitogen-activated protein kinase/Nemo-like kinase (MAPK/NLK)-dependent nuclear export of phosphorylated POP-1 from the nucleus of the cell in which the pathway is active, leading to a reduction in POP-1 levels in the nucleus of this daughter cell. Reciprocal asymmetry with respect to the coactivator SYS-1 leads to the activation of target genes in this cell. In contrast, POP-1 targets are repressed in the sister cell nucleus due to a reduced level of SYS-1; this catalyses asymmetric cell division, which is considered a fundamental tenet of *C. elegans* embryonic and larval development. 

Members of the Notch gene family (Figure 2A) encode receptors that transduce extracellular signals for specifying cell fates and tissue morphogenesis [94]. They are highly conserved throughout the animal kingdom and are single copy in most protostomes, including flies [95,96]. *C. elegans* has two paralogous Notch-like receptor loci, lin-12 and glp-1.

Following the sequencing and assembly of 10 new Caenorhabditis genomes by Mark Blaxter and colleagues, it was found that the duplication of the Notch-like receptor was not common to the genus, but restricted to species in the Elegans supergroup [22]. Basal taxa in the Drosophilae supergroup, such as C. monodelphis and C. plicata, encode only a single Notch-like receptor. Evidence for the further duplication of the glp-1 locus at the base of the Elegans supergroup was found in species belonging to the Japonica group, such as C. japonica and C. sulstoni, which have retained both copies of the glp-1-like gene, and therefore encode three Notch-like receptor genes in total. As such, what was previously assumed to be an evolutionarily constrained locus in the animal kingdom is actually a dynamic gene family in the Caenorhabditis genus. It is likely that the Notch receptor family behaves similarly in many metazoan taxa, but at present, evolutionary biologists cannot assess this due to the poor genomic characterisation of many animal groups. This highlights the special relevance of Caenorhabditis systems for understanding the complexities of gene family evolution today. 

Even before the genome of *C. elegans* was published, lin-12 and glp-1 were known to have redundant roles during embryogenesis [97]. Unlike either single mutant, double mutants lacking zygotic lin-12 and glp-1 activity die soon after hatching. Later in development, GLP-1/Notch signalling is required for germline differentiation [98], and LIN-12/Notch signalling is essential for vulval organogenesis [99]. The divergence of lin-12 and glp-1 is less clear cut when considering that they are able to substitute for one another during post-embryonic development [97,100]. Thus, it is thought that their divergent roles in late development may rely on differences in spatiotemporal expression [97].

The four Notch receptors in mice and humans are ohnologues. The redundancy of Notch1 and Notch2 in the immune response upon infection with *Leishmania major* has been recently recorded [101], but their relationship during development remains unelucidated. The expression domains of each Notch receptor are partially overlapping in mice [102]. Whether or not the mammalian Notch receptors have functionally diverged in development is unclear, but the maintenance of complete genetic redundancy over such long periods of evolutionary time is generally considered to be unstable and unlikely [11,12]. Therefore, it is striking that mice and nematodes have independently-derived Notch receptor duplications that have remained redundant through 80 million years of evolution; greater understanding of this may reveal important underlying properties of Notch signalling.

## 5. Conclusions

We argue that the field of nematode evolutionary developmental biology is now at a critical turning point because of the confluence of comparative developmental biology and comparative genomics. The characterisation of developmental variation in the nematode phylum is enabling new questions to be asked about the evolution of development. For example, why does selection act differently on different developmental events? Why are radical patterns of gene duplication and loss sometimes permitted during evolution? Why should gene toolkit comparisons go beyond analysis of copy number? The comparative developmental studies that enable these questions to be answered are the products of rich decades of research using *C. elegans*, a debt that evolutionary developmental biology using other nematode species is just beginning to repay. The wealth of genomic data gathered from these species is permitting the elucidation of gene family evolution in unprecedented phylogenetic detail. Whilst this work often depicts worms as animal oddities, meaning they have historically been understudied by evolutionary developmental biologists, these taxon-specific patterns of gene toolkit divergence are shedding light on the underlying principles of molecular evolution that apply to worms as well as all other metazoans.

## Abbreviations

Mb	MegaBase
*mab-5*	MAle aBnormal 5
Ceh (gene class)	*C. elegans* Homeobox
*tbx-43*	T-BoX 43
RNAi	RNA Interference
PAR proteins	PARtitioning proteins
TGF-β	Transforming Growth Factor β
ORFs	Open Reading Frames
Gli	GLIoma-associated oncogene homologue
Ci	Cubitus Interruptus
*tra-1*	TRAnsformer XX animals transformed into males
Lin (gene class)	LINeage defective
*egl-5*	Egg Laying Defective 5
*php-3*	Posterior Hox gene Paralogue 3
*nob-1*	kNOB-like posterior (NO Backside) 1
Cdx	CauDal Type HomeoboX
*pal-1*	Posterior ALae in Males 1
*sop-2*	Suppressor of Pal-1
SYS-1	SYmmetrical Sister cell hermaphrodite gonad defect
WRM-1	Worm aRMadillo
BAR-1	Beta-catenin/Armadillo Related
HMP-2	HuMPback (dorsal lumps)
TCF	T-Cell Factor
POP-1	POsterior Pharynx defect
*glp-1*	abnormal Germ Line Proliferation 1
BLAST	Basic Local Alignment Search Tool
